# (*E*)-2-(2-Hy­droxy-5-iodo­benzyl­idene)hydrazinecarboxamide

**DOI:** 10.1107/S1600536812010197

**Published:** 2012-03-17

**Authors:** Rahman Bikas, Samra Nikbakht Sardari, Seyed Sajjad Hosseini, Gholam Hossein Shahverdizadeh, Behrouz Notash

**Affiliations:** a Young Researchers Club, Tabriz Branch, Islamic Azad University, Tabriz, Iran; b Department of Chemistry, Ardabil Branch, Islamic Azad University, Ardabil, Iran; c Department of Chemistry, Faculty of Science, Tabriz Branch, Islamic Azad University, PO Box 1655, Tabriz, Iran; d Department of Chemistry, Shahid Beheshti University, G. C., Evin, Tehran, 1983963113, Iran

## Abstract

In the title mol­ecule, C_8_H_8_IN_3_O_2_, there is an intra­molecular O—H⋯N hydrogen bond between the hy­droxy group and the imine N atom, which generates an *S*(6) ring. In the crystal, the carbonyl O atom accepts two different N—H⋯O hydrogen bonds, which connect mol­ecules with two *R*_2_^2^(8) motifs.

## Related literature

For historical background to semicarbazones, see: Arapov *et al.* (1987 ▶); Pickart *et al.* (1983 ▶). For related structures see: Bikas *et al.* (2010 ▶, 2012*a* ▶,*b* ▶); Monfared *et al.* (2010*a* ▶). For background to the development of hydrazide derivatives for biological evaluation, see: Carvalho *et al.* (2008 ▶). For catalytic applications of aroylhydrazones, see: Monfared *et al.* (2010*b* ▶). For a similiar structure, see: Abboud *et al.* (1995 ▶).
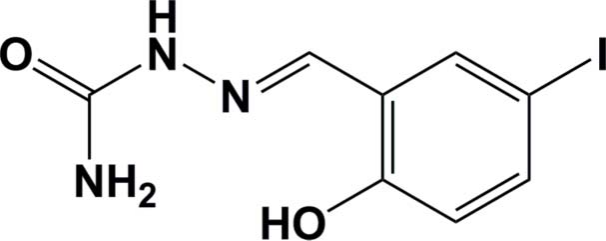


## Experimental

### 

#### Crystal data

C_8_H_8_IN_3_O_2_
*M* *_r_* = 305.07
Monoclinic, 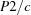

*a* = 9.1066 (18) Å
*b* = 7.6277 (15) Å
*c* = 14.375 (3) Å
β = 95.31 (3)°
*V* = 994.3 (3) Å^3^
*Z* = 4
Mo *K*α radiation
μ = 3.20 mm^−1^
*T* = 120 K
0.25 × 0.13 × 0.12 mm

#### Data collection

Stoe IPDS 2T diffractometer
Absorption correction: numerical (shape of crystal determined optically; *X-RED32* and *X-SHAPE*, Stoe & Cie, 2005 ▶) *T*_min_ = 0.502, *T*_max_ = 0.700
10438 measured reflections
2686 independent reflections
2362 reflections with *I* > 2σ(*I*)
*R*_int_ = 0.041

#### Refinement

*R*[*F* ^2^ > 2σ(*F* ^2^)] = 0.029
*wR*(*F* ^2^) = 0.056
*S* = 1.13
2686 reflections
143 parameters
1 restraint
H atoms treated by a mixture of independent and constrained refinement
Δρ_max_ = 0.75 e Å^−3^
Δρ_min_ = −0.71 e Å^−3^

### 

Data collection: *X-AREA* (Stoe & Cie, 2005 ▶); cell refinement: *X-AREA*; data reduction: *X-AREA*; program(s) used to solve structure: *SHELXS97* (Sheldrick, 2008 ▶); program(s) used to refine structure: *SHELXL97* (Sheldrick, 2008 ▶); molecular graphics: *ORTEP-3 for Windows* (Farrugia, 1997 ▶); software used to prepare material for publication: *WinGX* (Farrugia, 1999 ▶).

## Supplementary Material

Crystal structure: contains datablock(s) I, global. DOI: 10.1107/S1600536812010197/vm2154sup1.cif

Structure factors: contains datablock(s) I. DOI: 10.1107/S1600536812010197/vm2154Isup2.hkl

Supplementary material file. DOI: 10.1107/S1600536812010197/vm2154Isup3.cml

Additional supplementary materials:  crystallographic information; 3D view; checkCIF report

## Figures and Tables

**Table 1:** Hydrogen-bond geometry (Å, °)

*D*—H⋯*A*	*D*—H	H⋯*A*	*D*⋯*A*	*D*—H⋯*A*
N3—H3*B*⋯O2^i^	0.81 (4)	2.13 (4)	2.920 (3)	163 (3)
N2—H2⋯O2^ii^	0.80 (4)	2.00 (4)	2.800 (3)	176 (3)
O1—H1⋯N1	0.84 (2)	1.88 (3)	2.628 (3)	147 (4)

Symmetry codes: (i) 
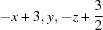
; (ii) 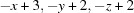.

**Fig. 1.**
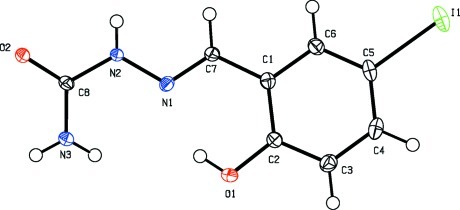
The molecular structure of the title compound. Displacement ellipsoids are drawn at the 50% probability level.

**Fig. 2.**
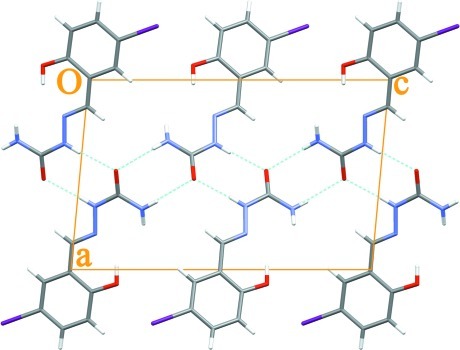
The packing diagram of the title compound showing intermolecular hydrogen bonds as blue dashed lines.

## supplementary crystallographic information

### Comment

Semicarbazone compounds are derived from the condensation of carbonyl compounds and semicarbazides. This class are important tridentate O, N, O-donor ligands. As biologically active compounds, semicarbazones find application in the treatment of diseases such as anti-tumor, tuberculosis, leprosy and mental disorder. Furthermore, semicarbazone have wide spread applications in fields such as coordination chemistry, bioinorganic chemistry, and in magnetic, electronic, nonlinear optically active and fluorescent compounds. Also semicarbazone metal complexes seem to be a good candidate for catalytic oxidation studies because of their resist to oxidation (Monfared *et al.*, 2010*b*).

As part of our studies on the synthesis and characterization of hydrazone derivatives (Bikas *et al.*, 2010; Bikas *et al.*, 2012*a*,*b*), we report here the crystal structure of (*E*)-2-(2-hydroxy-5-iodobenzylidene)hydrazinecarboxamide (Fig.1). Bond distances are in the normal range for similar hydrazone compounds (Abboud *et al.*, 1995). The molecule is approximately planar, with an r.m.s. deviation from the mean plane through all 14 non-H atoms of 0.181 (2) Å. The dihedral angle between the phenyl ring plane and the least-squares plane through the N3—C8—O2—N2 unit is 14.00 (13)°. In the crystal structure of the title compound, the molecule adopts an *E* configuration with respect to the C7=N1 bond. In the crystal structure of the title compound, there is an intramolecular O—H···N hydrogen bonding between the hydroxyl group and imine nitrogen atom. The carbonyl group forms two different intermolecular N—H···O hydrogen bonds parallel to *ac-* plane which connects molecules with two *R*_2_^2^(8) motifs (Table 1, Fig. 2).

### Experimental

For preparing the title compound a methanol (10 ml) solution of 2-hydroxy-5-iodobenzaldehyde (1.5 mmol) was added drop-wise to a methanol solution (10 ml) of semicarbazide (1.5 mmol), and the mixture was refluxed for 3 h. The solution was then evaporated on a steam bath to 5 ml and cooled to room temperature. The light-yellow precipitates of the title compound were separated and filtered off, washed with 3 ml of cooled methanol and then dried in air. Colorless crystals were obtained from its methanol solution by slow solvent evaporation. Yield: 92%. IR (cm^-1^): 3464 (m, O—H), 3176 (m, broad, N—H), 1699 (*vs*, C=O), 1594 (s, C=N), 1463 (*s*), 1340 (*m*), 1259 (*vs*), 1187 (*s*), 1072 (*m*), 942 (*vs*), 893 (*m*), 818 (*m*), 769 (*vs*), 682 (*m*), 613 (*m*), 572 (*vs*), 517 (*s*), 522 (*m*), 472 (*vs*), 427 (*vs*).

### Refinement

The hydrogen atoms of the N—H and O—H groups were found in a difference Fourier map and refined isotropically with a distance restraint to 0.84 Å for the O—H group. All other H atoms were positioned geometrically and refined as riding atoms with C—H = 0.95 Å, *U*_iso_(H) = 1.2*U*_eq_(C) aromatic and imine H atoms.

### Crystal data

**Table:**

C_8_H_8_IN_3_O_2_	*F*(000) = 584
*M**_r_* = 305.07	*D*_x_ = 2.038 Mg m^−^^3^
Monoclinic, *P*2/*c*	Mo *K*α radiation, λ = 0.71073 Å
Hall symbol: -P 2yc	Cell parameters from 2686 reflections
*a* = 9.1066 (18) Å	θ = 2.7–29.2°
*b* = 7.6277 (15) Å	µ = 3.20 mm^−^^1^
*c* = 14.375 (3) Å	*T* = 120 K
β = 95.31 (3)°	Block, colorless
*V* = 994.3 (3) Å^3^	0.25 × 0.13 × 0.12 mm
*Z* = 4	

### Data collection

**Table:**

Stoe IPDS 2T diffractometer	2686 independent reflections
Radiation source: fine-focus sealed tube	2362 reflections with *I* > 2σ(*I*)
Graphite monochromator	*R*_int_ = 0.041
Detector resolution: 0.15 mm pixels mm^-1^	θ_max_ = 29.2°, θ_min_ = 2.7°
rotation method scans	*h* = −12→12
Absorption correction: numerical (shape of crystal determined optically; *X-RED32* and *X-SHAPE*, Stoe & Cie, 2005)	*k* = −10→10
*T*_min_ = 0.502, *T*_max_ = 0.700	*l* = −18→19
10438 measured reflections	

### Refinement

**Table:**

Refinement on *F*^2^	Primary atom site location: structure-invariant direct methods
Least-squares matrix: full	Secondary atom site location: difference Fourier map
*R*[*F*^2^ > 2σ(*F*^2^)] = 0.029	Hydrogen site location: inferred from neighbouring sites
*wR*(*F*^2^) = 0.056	H atoms treated by a mixture of independent and constrained refinement
*S* = 1.13	*w* = 1/[σ^2^(*F*_o_^2^) + (0.0208*P*)^2^ + 1.0932*P*] where *P* = (*F*_o_^2^ + 2*F*_c_^2^)/3
2686 reflections	(Δ/σ)_max_ = 0.001
143 parameters	Δρ_max_ = 0.75 e Å^−^^3^
1 restraint	Δρ_min_ = −0.71 e Å^−^^3^

### Special details

**Table:**

Geometry. All e.s.d.'s (except the e.s.d. in the dihedral angle between two l.s. planes) are estimated using the full covariance matrix. The cell e.s.d.'s are taken into account individually in the estimation of e.s.d.'s in distances, angles and torsion angles; correlations between e.s.d.'s in cell parameters are only used when they are defined by crystal symmetry. An approximate (isotropic) treatment of cell e.s.d.'s is used for estimating e.s.d.'s involving l.s. planes.
Refinement. Refinement of *F*^2^ against ALL reflections. The weighted *R*-factor *wR* and goodness of fit *S* are based on *F*^2^, conventional *R*-factors *R* are based on *F*, with *F* set to zero for negative *F*^2^. The threshold expression of *F*^2^ > σ(*F*^2^) is used only for calculating *R*-factors(gt) *etc*. and is not relevant to the choice of reflections for refinement. *R*-factors based on *F*^2^ are statistically about twice as large as those based on *F*, and *R*- factors based on ALL data will be even larger.

### Fractional atomic coordinates and isotropic or equivalent isotropic displacement parameters (Å^2^)

**Table:**

	*x*	*y*	*z*	*U*_iso_*/*U*_eq_	
I1	0.71642 (2)	0.52615 (2)	1.210107 (13)	0.02620 (6)	
O2	1.52011 (18)	1.0591 (2)	0.88088 (11)	0.0160 (3)	
N2	1.3349 (2)	0.9116 (3)	0.93933 (14)	0.0149 (4)	
C8	1.3955 (2)	0.9896 (3)	0.86612 (16)	0.0137 (4)	
N3	1.3205 (2)	0.9864 (3)	0.78198 (15)	0.0166 (4)	
N1	1.1902 (2)	0.8602 (3)	0.93061 (14)	0.0134 (4)	
O1	0.9242 (2)	0.8354 (3)	0.84348 (13)	0.0217 (4)	
C1	0.9856 (3)	0.7452 (3)	1.00333 (17)	0.0139 (4)	
C2	0.8840 (3)	0.7661 (3)	0.92413 (17)	0.0150 (4)	
C7	1.1398 (3)	0.7979 (3)	1.00415 (16)	0.0137 (4)	
H7	1.2036	0.7860	1.0599	0.016*	
C6	0.9357 (3)	0.6755 (3)	1.08517 (17)	0.0165 (5)	
H6	1.0028	0.6609	1.1393	0.020*	
C4	0.6899 (3)	0.6494 (3)	1.00927 (19)	0.0191 (5)	
H4	0.5895	0.6172	1.0115	0.023*	
C3	0.7373 (3)	0.7178 (3)	0.92780 (18)	0.0187 (5)	
H3	0.6691	0.7319	0.8742	0.022*	
C5	0.7893 (3)	0.6278 (3)	1.08752 (18)	0.0173 (5)	
H2	1.380 (4)	0.917 (4)	0.990 (3)	0.022 (8)*	
H3A	1.244 (4)	0.929 (5)	0.773 (3)	0.029 (9)*	
H3B	1.359 (4)	1.028 (5)	0.738 (3)	0.028 (9)*	
H1	1.016 (2)	0.849 (5)	0.849 (3)	0.037 (10)*	

### Atomic displacement parameters (Å^2^)

**Table:**

	*U*^11^	*U*^22^	*U*^33^	*U*^12^	*U*^13^	*U*^23^
I1	0.03341 (10)	0.02256 (9)	0.02507 (9)	−0.00405 (7)	0.01569 (7)	0.00293 (7)
O2	0.0139 (7)	0.0241 (10)	0.0102 (8)	−0.0040 (6)	0.0015 (6)	−0.0006 (6)
N2	0.0121 (9)	0.0243 (10)	0.0081 (9)	−0.0052 (8)	0.0003 (7)	0.0002 (8)
C8	0.0143 (9)	0.0174 (12)	0.0095 (9)	0.0005 (8)	0.0019 (8)	−0.0004 (8)
N3	0.0153 (9)	0.0243 (11)	0.0101 (9)	−0.0040 (8)	0.0007 (7)	0.0005 (8)
N1	0.0112 (9)	0.0159 (9)	0.0132 (9)	−0.0012 (7)	0.0011 (7)	−0.0023 (7)
O1	0.0150 (9)	0.0375 (11)	0.0121 (8)	−0.0015 (7)	−0.0009 (7)	0.0058 (7)
C1	0.0149 (11)	0.0130 (12)	0.0141 (10)	−0.0002 (8)	0.0032 (8)	−0.0014 (8)
C2	0.0147 (11)	0.0174 (11)	0.0131 (11)	−0.0005 (8)	0.0020 (8)	−0.0005 (9)
C7	0.0130 (10)	0.0171 (11)	0.0109 (10)	−0.0006 (8)	0.0010 (8)	−0.0011 (8)
C6	0.0176 (11)	0.0180 (12)	0.0140 (11)	0.0003 (9)	0.0030 (9)	0.0010 (9)
C4	0.0147 (11)	0.0188 (12)	0.0249 (13)	−0.0037 (9)	0.0070 (9)	−0.0054 (10)
C3	0.0149 (11)	0.0231 (13)	0.0181 (12)	0.0001 (9)	0.0017 (9)	−0.0031 (9)
C5	0.0204 (11)	0.0137 (11)	0.0194 (12)	−0.0014 (8)	0.0106 (9)	0.0005 (9)

### Geometric parameters (Å, º)

**Table:**

I1—C5	2.089 (2)	C1—C6	1.404 (3)
O2—C8	1.253 (3)	C1—C2	1.408 (3)
N2—C8	1.369 (3)	C1—C7	1.460 (3)
N2—N1	1.369 (3)	C2—C3	1.391 (3)
N2—H2	0.80 (4)	C7—H7	0.9500
C8—N3	1.333 (3)	C6—C5	1.385 (3)
N3—H3A	0.82 (4)	C6—H6	0.9500
N3—H3B	0.81 (4)	C4—C5	1.387 (4)
N1—C7	1.282 (3)	C4—C3	1.387 (4)
O1—C2	1.355 (3)	C4—H4	0.9500
O1—H1	0.841 (18)	C3—H3	0.9500
			
C8—N2—N1	120.5 (2)	C3—C2—C1	120.0 (2)
C8—N2—H2	118 (2)	N1—C7—C1	120.9 (2)
N1—N2—H2	120 (2)	N1—C7—H7	119.6
O2—C8—N3	122.8 (2)	C1—C7—H7	119.6
O2—C8—N2	118.5 (2)	C5—C6—C1	120.4 (2)
N3—C8—N2	118.7 (2)	C5—C6—H6	119.8
C8—N3—H3A	121 (3)	C1—C6—H6	119.8
C8—N3—H3B	118 (3)	C5—C4—C3	119.9 (2)
H3A—N3—H3B	120 (4)	C5—C4—H4	120.0
C7—N1—N2	116.5 (2)	C3—C4—H4	120.0
C2—O1—H1	108 (3)	C4—C3—C2	120.4 (2)
C6—C1—C2	118.8 (2)	C4—C3—H3	119.8
C6—C1—C7	118.9 (2)	C2—C3—H3	119.8
C2—C1—C7	122.3 (2)	C6—C5—C4	120.4 (2)
O1—C2—C3	118.2 (2)	C6—C5—I1	120.0 (2)
O1—C2—C1	121.8 (2)	C4—C5—I1	119.59 (17)
			
N1—N2—C8—O2	169.1 (2)	C2—C1—C6—C5	0.2 (4)
N1—N2—C8—N3	−11.9 (3)	C7—C1—C6—C5	178.5 (2)
C8—N2—N1—C7	−175.9 (2)	C5—C4—C3—C2	−0.3 (4)
C6—C1—C2—O1	178.8 (2)	O1—C2—C3—C4	−178.8 (2)
C7—C1—C2—O1	0.6 (4)	C1—C2—C3—C4	0.1 (4)
C6—C1—C2—C3	−0.1 (4)	C1—C6—C5—C4	−0.4 (4)
C7—C1—C2—C3	−178.3 (2)	C1—C6—C5—I1	−179.51 (18)
N2—N1—C7—C1	177.8 (2)	C3—C4—C5—C6	0.5 (4)
C6—C1—C7—N1	179.1 (2)	C3—C4—C5—I1	179.59 (19)
C2—C1—C7—N1	−2.6 (4)		

### Hydrogen-bond geometry (Å, º)

**Table:**

*D*—H···*A*	*D*—H	H···*A*	*D*···*A*	*D*—H···*A*
N3—H3*B*···O2^i^	0.81 (4)	2.13 (4)	2.920 (3)	163 (3)
N2—H2···O2^ii^	0.80 (4)	2.00 (4)	2.800 (3)	176 (3)
O1—H1···N1	0.84 (2)	1.88 (3)	2.628 (3)	147 (4)

Symmetry codes: (i) −*x*+3, *y*, −*z*+3/2; (ii) −*x*+3, −*y*+2, −*z*+2.
